# Supplementary material for: The interactome of intact mitochondria by cross-linking mass spectrometry provides evidence for coexisting respiratory supercomplexes
Source: Mol Cell Proteomics. 2017 Dec 8;17(2):216–32. doi: 10.1074/mcp.RA117.000470 (PMC5795388; doi:10.1074/mcp.RA117.000470)
Supplement: Supplemental Data [file supp_RA117.000470_133922_0_supp_23978_fzffwf.zip › spectra_annotation/mito_DR_spectra_annotation/406-2-89-1-130-1.pdf]

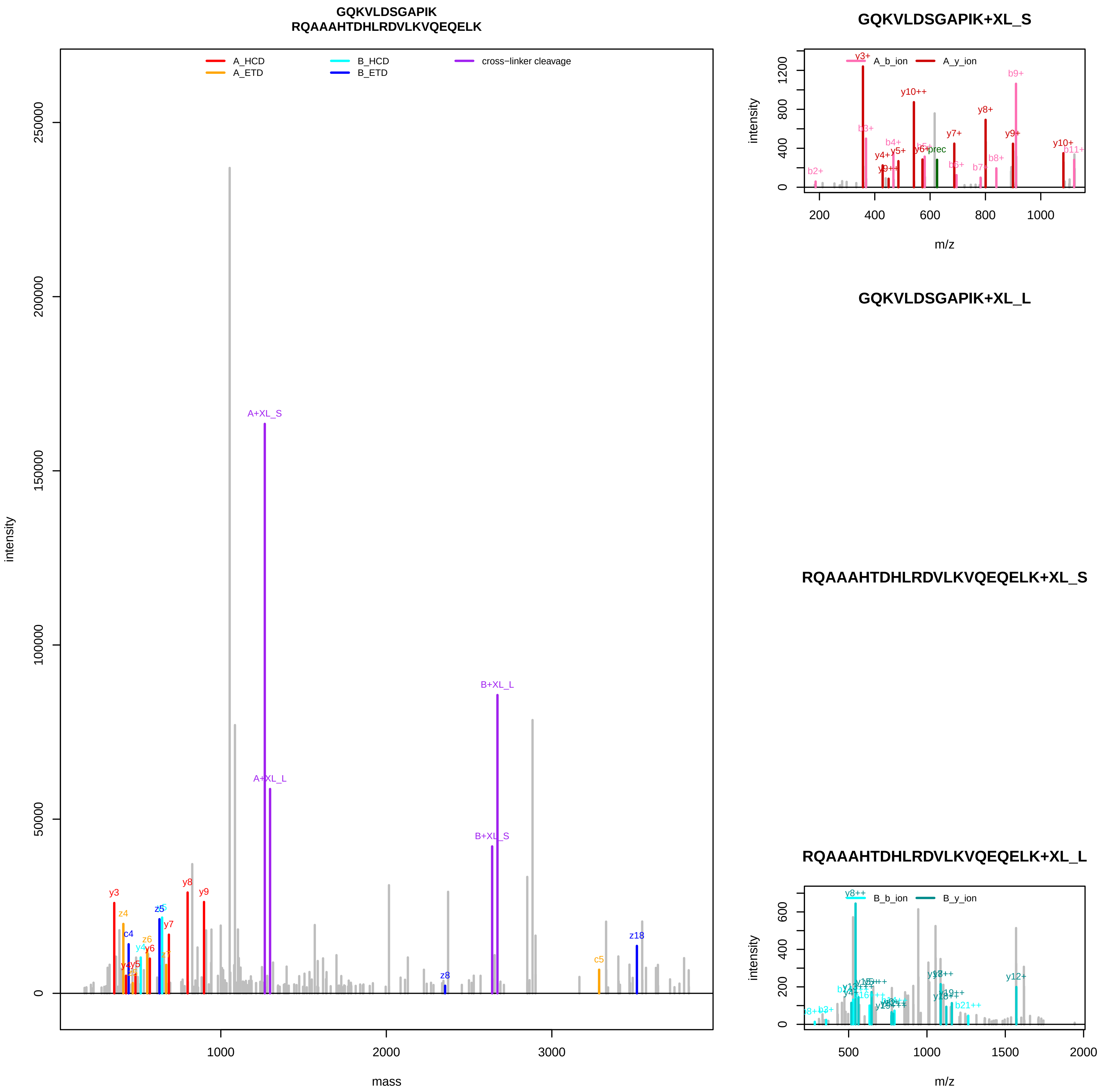
GQKVLDSGAPIK
RQAAAHTDHLRDVLKVQEQELK
A_HCD
A_ETD
B_HCD
B_ETD
cross−linker cleavage
A+XL_S
A+XL_L
B+XL_L
B+XL_S
intensity
mass
0
50000
100000
150000
200000
250000
1000
2000
3000
y3
z4
c4
y4
y5
z6
y6
z5
y7
y8
y9
z8
c5
z18
GQKVLDSGAPIK+XL_S
A_b_ion
A_y_ion
y3+
b9+
y10++
b3+
y8+
y7+
b4+
y9+
y4+
y5+
y6+
prec
y10+
b11+
b2+
b6+
b7+
b8+
0
400
800
1200
200
400
600
800
1000
m/z
GQKVLDSGAPIK+XL_L
RQAAAHTDHLRDVLKVQEQELK+XL_S
RQAAAHTDHLRDVLKVQEQELK+XL_L
B_b_ion
B_y_ion
y8++
y12+
b21++
0
200
400
600
500
1000
1500
2000
